# Resonance Modes of Water Drops Pinned to a Vibrating Rectangular Post

**DOI:** 10.3390/mi15050634

**Published:** 2024-05-09

**Authors:** Paolo Sartori, Davide Ferraro, Matteo Pierno, Giampaolo Mistura

**Affiliations:** Department of Physics and Astronomy, University of Padua, Via Marzolo 8, 35131 Padua, Italy; paolo.sartori.3@unipd.it (P.S.); davide.ferraro@unipd.it (D.F.); matteo.pierno@unipd.it (M.P.)

**Keywords:** acoustofluidics, wetting, anisotropic wetting, normal modes, microfabrication

## Abstract

We studied the effects of vertical vibrations on a water drop that was pinned to the sharp edges of a rectangular post. By varying the frequency and amplitude of the vertical displacement, distinct resonance peaks were observed using a simple optical technique. The vibrational spectra of the first two modes exhibited two closely spaced peaks, which corresponded to standing waves that exist along the major and minor contour lengths of the drops. The values of the resonance frequencies can be explained rather well by a simple model, which was originally proposed for axially symmetric drops.

## 1. Introduction

The wetting of a surface by a liquid is an everyday phenomenon that depends on both surface chemistry and surface morphology [1]. The presence of surface microstructures can enhance or inhibit the inhomogeneous distribution of a liquid in certain directions [2,3,4,5], giving rise to anisotropic droplet shapes [6] and interfacial instabilities [7]. In this case, anisotropy is due to the pinning of the contact line to the sharp edges of the asperities. Anisotropic wetting behavior is exhibited by many natural and synthetic surfaces. For example, the surfaces of many plants and animals are patterned by linear microstructures that can guide the motion of water droplets in well-defined directions [8]. Inspired by this rich phenomenology, many studies of anisotropic wetting have been conducted using biomimetic surfaces [9,10,11,12,13,14]. Particular attention has been devoted to the evolution of the shape of a liquid drop confined to the upper face of a rectangular post as its volume *Ω* increases. It is found that for small *Ω*, the liquid assumes a shape with a uniform cross section, while for large *Ω*, the liquid shows a central bulge [15]. Increasing the volume of the water drop leads to a morphological transition between the two shapes, which is observed for different geometries of the post [16,17,18,19].

The morphology transition from a flat channel into a localized bulge can also be induced by vertical vibrations of suitable amplitude [20]. Vibrations have been successfully used to actuate drops on surfaces regardless of the liquid properties [21,22,23], even against the action of gravity [24,25]. Vibrations can also induce the excitation of normal modes of the droplet, which can be used as a tool to measure the surface tension and viscosity of small droplets [26,27] or to micro-nebulize a liquid sample for biochemical analysis [28].

**Figure 1:**
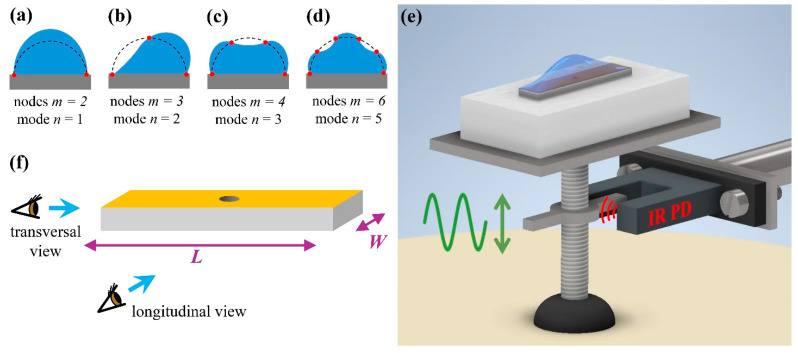
Schematic representation of the drop profiles for the modes *n* = 1 (**a**), *n* = 2 (**b**), *n* = 3 (**c**), and *n* = 5 (**d**). The dashed lines indicate the static contour. The nodes are highlighted with red circles. (**e**) Sketch of the rectangular post attached to the shaft of the shaker. The vertical displacement of the shaft was measured with the infrared photodetector (IR PD). (**f**) Geometry of the rectangular post. Water was infused into the drop through the central hole. The post top face in contact with water was covered with a hydrophilic gold layer, whereas the vertical walls were hydrophobic.

In general, the determination of the normal modes of a drop subject to vertical vibrations is studied in the two opposite limits of a fixed contact line and of a freely moving contact line: the former modes occur at a low amplitude of vibrations, whereas the latter modes appear above a threshold amplitude [29]. In the case of vibrated drops deposited on a homogeneous surface, axisymmetric surface waves of discrete frequencies are observed [30]. The different modes are stationary surface waves that exhibit a complicated three-dimensional wave pattern. Extensive numerical simulations are required to address the problem with gravity [31], and no analytical expressions are available to determine the resonance spectrum. To simplify the problem, Noblin et al. [30] considered the waves as one dimensional. In general, the drop contour can present *m* nodes (m=2, 3, 4…), that is, characteristic points that do not move during periodic vibrations. The mean distance between two consecutive nodes along the drop contour is the corresponding pseudo wavelength $\lambda_{m}$. If the drop contact line is fixed, one obtains the geometric relation $\left(m-1\right)\lambda_{m}/2=l$, where l represents the length of the drop contour at equilibrium [30]. In other words, these authors suggested that the resonant vibrational states of drops with pinned contact lines could be described by assuming that an integer number n=m−1 of half vibrational wavelengths fits along the contour length of the drops, where n=1, 2, 3… can be identified as the mode number [32]. Examples of the drop shapes expected for the lowest frequency modes are shown in Figure 1a–d. The *n* = 1 mode implies a volume change, and is then not allowed for an incompressible liquid. The mode *n =* 2 is the lowest vibration mode that conserves volume for sessile drops when the contact line remains pinned and can be excited by a lateral vibration due to the substrate (“rocking” motion) [33]. The remaining panels represent the first two frequency modes (*n =* 3 and *n =* 4) that can be excited by vertical vibrations. 

The pseudo wave vector values $q_{n}\left(\Omega \right)$, which correspond to the mode of order *n* and to a drop of a given volume Ω, can be calculated from

(1) $$q_{n}\left(\Omega \right)=\frac{2\pi }{\lambda_{n}}=\frac{\pi n}{l}$$

The general dispersion relation between the frequency *f* and the wave vector *q* of 1D capillary-gravity waves on a liquid bath of depth *h* assumed to be less than the capillary length of the liquid ($l_{cap}=\sqrt{\gamma /\varrho g}\sim$2.7 mm for water) is given by [34]:

(2) $$f^{2}=\frac{1}{4\pi^{2}}\left(gq+\frac{\gamma }{\varrho }q^{3}\right)\tanh\left(qh\right)$$

where γ is the surface tension and ϱ is the mass density of the liquid, respectively, and g is the acceleration due to gravity. In their analysis, Noblin et al. [30] assumed for the wave vector the approximate Equation (1) and for *h*, the mean height of the drop profile $\bar{h}$ defined by

(3) $$\bar{h}=\frac{\Omega }{\Sigma }$$

where Σ is the wet area, that is, the area of the surface that is in contact with the drop. In other words, the resonance frequencies of the various normal modes can be determined by the following equation:

(4) $$f_{n}^{2}=\frac{1}{4\pi^{2}}\left(gq_{n}+\frac{\gamma }{\varrho }q_{n}^{3}\right)\tanh\left(q_{n}\bar{h}\right)$$

Despite its simplicity, this analysis was found to describe the experimental data rather well in the case of axially symmetric sessile drops of different volumes [30]. It was also successful in describing the dependence of the resonance frequency on the viscosity and contact angle of the drop [35,36]. If the drops are instead elongated along one direction, the vibrational spectra exhibit pairs of two nearby peaks. An extension of the model summarized by Equation (3) explains this observation as the result of the splitting of the fundamental vibration mode caused by the anisotropic wetting of the drop: the two closely spaced vibrational frequencies correspond to standing wave states that exist along the profile lengths related to the major and minor drop axes of the ellipsoidal drop [32].

In this work, we extend these studies to liquid drops, whose contact line is rectangular rather than circular or elliptical. The contact line was pinned to the sharp edges of a rectangular post that underwent vertical oscillations. Distinct resonance peaks were observed, which corresponded to the excitation of normal modes along the drop contour. Their values were accounted for rather well by a simple model, which was originally proposed for spherically symmetric drops [30]. The rest of this paper is organized as follows. Section 2 briefly describes the materials and experimental setups; we then present the measured resonance spectra and discuss the results in terms of the model of Noblin et al. [30].

## 2. Materials and Methods

### 2.1. Optofluidic Setup

The patterned sample was attached to the shaft of an electromagnetic shaker that could vibrate vertically, as shown in the schematic diagram in Figure 1e. The accessible frequency range was from 10 Hz to 10 kHz with a maximum vibrating amplitude A = 2.5 mm. An infrared photodetector (PD) was used to measure A in real-time with an estimated resolution close to 2 μm. This was calibrated by optically measuring the net displacement of the shaft. The shaker amplitude A decreases with the vibrating frequency *f*. Using a custom-made feedback circuit, it is also possible to sweep *f* while maintaining *A* constant over a frequency interval of approximately 500 Hz.

A liquid drop can be produced on the upper face of the rectangular post by connecting the central hole to a thin tube attached to a syringe pump (Harvard Apparatus, Holliston, MA, USA). A flow meter (Fluigent, Le Kremlin-bicêtre, France) was used to measure the drop volume Ω. The time evolution of the drop contour was recorded with a custom-made apparatus. Two LED sources (Phlox) could back-illuminate the drop from the two sides of the rectangular post. The drop contour was simultaneously viewed from the two orthogonal sides of the post with two high-resolution CCD cameras (Manta G-146B, Allied Vision Technologies, Stadtroda, Germany) equipped with 2× telecentric objectives (VS-TC2-110, VS Technology, Tokyo, Japan), as shown in Figure 1f. To neglect evaporation, the sample was enclosed in a transparent box with a controlled degree of humidity. Considering that a full acquisition run takes less than 5 min, we can confidently state that Ω remained constant during the measurement of the vibrational spectra. Further details on the setup have been reported elsewhere [20].

### 2.2. Fabrication of the Rectangular Posts

**Figure 2:**
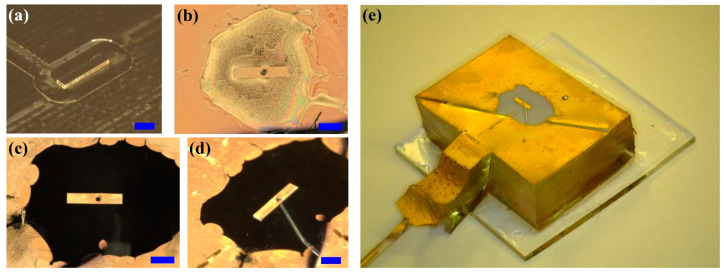
(**a**) Brass master copy of the individual post produced by micro-milling, to be used as a mold. (**b**) Gold layer on the PDMS post with vertical walls protected by NOA 61 adhesive. (**c**,**d**) Post with NOA 61 peeled off; the thin tube for water infusion can be observed through transparent PDMS in (**d**). All scale bars, indicated in blue, correspond to 1 mm. (**e**) Overview of the PDMS post glued to a microscope glass slide.

Special care was taken to fabricate rectangular posts with sharp edges and corners. Individual posts in polydimethilsiloxane (PDMS) with a rectangular cross section were manufactured by a double replica molding technique, following a similar procedure used in a previous study [15]. To ensure the planarity of the posts, micro-milling (Minitech Machinery Co., Norcross, GA, USA) was used for mold microfabrication, starting from a polished brass plate (see Figure 2a). The characteristic dimensions of the posts were: height h = 100 μm, length L = 2500 μm and width W = 500 μm; the corresponding aspect ratio is then l=L/W=5. The upper face of the PDMS post presented a through hole with a diameter ~150 μm in the center that allowed for the infusion of water on its surface (see Figure 2b–d).

Once produced, the PDMS posts are hydrophobic, with a contact angle θ_0_ = 110° for water. To exclusively modify the wettability of their upper faces, these were coated with a thin gold layer with a thickness of ~200 nm deposited by magneto-sputtering. Before the sputtering process, the vertical walls were covered with a UV curable optical adhesive, NOA 61, preserving them from gold deposition, as shown in Figure 2b. Right before each measurement, the gold surface was exposed to oxygen plasma (Diener electronic, Ebhausen, Germany), and then the NOA 61 coating was peeled off (see Figure 2c,d). As a result, the contact angle on the upper face was reduced to θ_0_~15° and remained stable for a couple of hours, while the vertical walls were hydrophobic. In this way, water was confined only at the top of the post. The volume of the water drop pinned to the edge of the post was controlled with a syringe pump. Figure 2e shows the individual post ready to be mounted on the shaker and connected to the syringe pump through a polyethylene tube.

## 3. Results and Discussion

**Figure 3:**
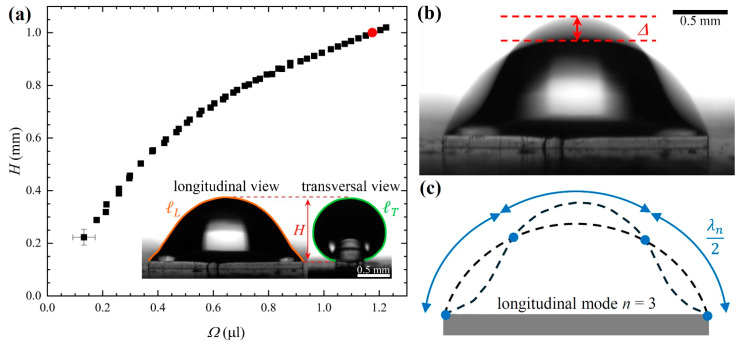
(**a**) Growth of the maximum height of drop H as a function of the drop volume Ω. Indicative error bars are only reported for the first point. The inset shows the equilibrium longitudinal and transversal images of the water drop corresponding to the red circle indicated in the graph. The drop contour lengths are highlighted in the two cases. (**b**) Longitudinal view of the vibrating water drop. The vertical peak-to-peak amplitude Δ of the drop contour is also indicated. (**c**) Schematic representation of the corresponding longitudinal mode: the nodes are indicated by blue circles. Dashed lines denote the static and instantaneous drop contours. The contour length between two consecutive nodes is the pseudo wavelength $\lambda_{n}$.

The graph in Figure 3a displays the maximum height H of the drop, measured from the upper face of the post, as a function of Ω. The inset displays the two orthogonal views of the drop corresponding to the red circle indicated on the graph. As expected, H continuously increased with Ω because the aspect ratio of the post was much smaller than the critical value $l_{crit}$ = 16, above which a discontinuous transition from a filament to the bulge state is predicted [15]. Furthermore, the use of a short post, that is, $l<l_{crit}$, guarantees that no morphology transition from the filament to the bulge state can be induced by applying vertical vibrations to the substrate [20]. Consequently, vibrating the post can only excite the normal modes of the drop pinned to its rectangular perimeter.

To study the normal modes of the water drop, we applied vertical sinusoidal vibrations to the sample, sweeping the frequency range at a constant amplitude. At each frequency f, we measured the vertical displacement of the vibrating drop contour along the symmetry axis by analyzing the shaded areas of the video frames, obtained by setting an acquisition time period for the video camera at least 10 times longer than the vibration period, so that each frame shows the superposition of many instantaneous profiles integrated over an extended time interval. The quantity Δ represents the height of the shaded area with respect to the equilibrium (black) contour, as indicated in Figure 3b, and was extracted from the individual frames using the free software ImageJ 1.54f. To ensure that the value of Δ was detectable at frequencies far from the resonance peaks, for each mode *n*, the frequency scan was performed with a different constant amplitude $A\left(f_{n}\right)$. The schematic diagram of Figure 3c highlights the corresponding static and instantaneous profiles, together with the indication of the nodes and the pseudo wavelength $\lambda_{n}$. Before each measurement, the drop was prepared by infusing water onto the rectangular post until a maximum height *H* = 1 mm was reached.

**Figure 4:**
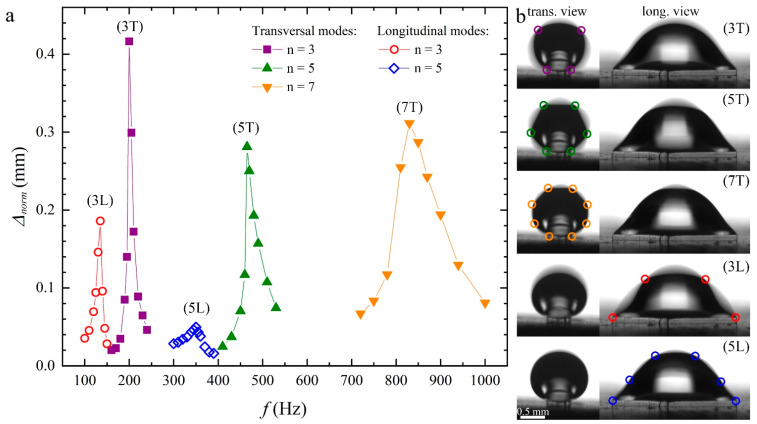
(**a**) Resonance peaks of water drops of volume Ω=1.17 μL deposited on the upper face of a post that undergoes vertical oscillations of constant amplitude. The vertical axis refers to the normalized vertical displacement $\Delta_{norm}$. The label beside each peak identifies the resonance mode. (**b**) Transversal (T) and longitudinal (L) normal modes observed from front and side views. Oscillation nodes are highlighted by colored circles. The colors correspond to the data in panel (**a**).

The graph in Figure 4a reports the resonance curves with the lowest frequencies. They refer to the same water volume Ω=1.17 μL, which corresponds to a maximum height of the static drop H=1 mm.

The *y*-axis reports the vertical displacement normalized to the vertical acceleration according to: $\Delta_{norm}=\Delta a_{ref}\left(f_{n}\right)/a\left(f_{n}\right)$, where $a\left(f_{n}\right)={\left(2\pi f_{n}\right)}^{2}A\left(f_{n}\right)$ is the acceleration corresponding to the resonance mode *n* and $a_{ref}\left(f_{n}\right)={\left(2\pi f_{n}\right)}^{2}A_{ref}$ is the reference acceleration corresponding to the amplitude $A_{ref}$, used to scan the lowest frequency mode. Each curve was identified with a label that reported the mode number *n* followed by the letter L (T), which stands for longitudinal (transversal). As expected, the vibrational spectra of the first two modes exhibited two closely spaced peaks, which corresponded to standing waves that exist along the major and minor contour lengths of the drops [32]. This interpretation was confirmed by the analysis of the two corresponding orthogonal views, which are shown in the (b) panels (see also Supplementary Video S1 taken at about 20 fps and with an exposure time of about 20 ms). In the different snapshots, the nodes are highlighted with empty circles. The modes were then labelled T (L) if the nodes appeared in the transversal (longitudinal) views. Only odd modes were observed because the drops underwent vertical vibrations. Instead, even modes (“rocking” modes) could only be excited by lateral vibrations.

The experimental resonance values are listed in Table 1 and can be compared with the predictions of the simple model expressed by Equation (4). The lengths of the transversal and longitudinal contours of the drops were $l_{T}=3.16\pm 0.05$ mm and $l_{L}=3.60\pm 0.05$ mm, respectively, as deduced by image processing of the two static contours. Similarly, the average height $\bar{h}$ was calculated by numerical integration of the two static images. More precisely, the average heights ${\bar{h}}_{T}$ and ${\bar{h}}_{L}$ of the transversal and longitudinal cross-section of the drop, respectively, calculated from the images as

(5) $${\bar{h}}_{T,L}=\frac{1}{\Sigma_{T,L}}\int_{0}^{H}z\rho_{T,L}\left(z\right)dz$$

where $\Sigma_{T,L}$ is the area of the cross-section and $\rho_{T,L}\left(z\right)$ is the width of the cross-section at the height *z*. The values obtained were ${\bar{h}}_{T}=0.48\pm 0.02$ mm and ${\bar{h}}_{L}=0.37\pm 0.02$ mm. In previous studies, the average height was calculated analytically assuming a semicircular contour [36,37,38].

The resonance frequencies for the different modes can then be derived from Equation (4), and the calculated values are listed in Table 1. The good agreement with the corresponding experimental values confirms that the simple model of Noblin et al. [30] also provides an accurate description in the extreme case of elongated drops that present a rectangular contact line. For a more precise estimate, the common procedure is to introduce a geometric factor α. This empirical factor is usually determined by studying the evolution of the resonance frequency with the drop volume [37,38]. The resonance frequency of a vibrating mode decreases with the volume of the drop volume according to $f=\alpha /\sqrt{\Omega }$, as it can be easily derived from Equations (1) and (4) under the assumption that the length of the drop contour scales as $l\sim \sqrt[{3}]{\Omega }$ (see e.g., Ref. [38]). By measuring the variation of f vs. $\sqrt{\Omega }$, the geometric factor can be empirically determined. However, this requires that the overall shape of the drop including contact angles do not vary with volume. This is straightforward on a flat, homogeneous surface where the contact line is a circle, and the contact angle does not vary with *Ω* and $\sqrt[{3}]{\Omega }<l_{cap}$ (see e.g., Ref. [38]) or for a surface patterned with narrow linear stripes where the contact line is an ellipse and the contact angles along the two main axes do not vary with *Ω* (see e.g., Ref. [32]). In our case, maintaining a rectangular contact line requires the fabrication of a series of rectangular posts with hydrophobic vertical faces and hydrophilic upper faces, all of which have the same aspect ratio but different sizes. This can be undertaken, but it requires much more effort. The analysis we present suggests that this calibration is not necessary, and that it is possible to evaluate the resonance modes once the drop geometry is known.

## 4. Conclusions

We reported on the detection of the first resonance modes of confined water drops that undergo vertical vibrations. The drop contact line was pinned to the contour of a rectangular post fabricated by double replica molding of a master obtained by micro-milling. The vibrational spectra of the first two modes exhibited two closely spaced peaks, which a direct visual analysis carried out with video cameras associated with the excitation of standing waves along the major and minor contour lengths of the drops. The values of the resonance frequencies could be explained rather well by a simple model, which was originally proposed for axially symmetric drops. Our results thus also extend the applicability of this model to highly anisotropic drops.

## Figures and Tables

**Table 1:** Resonance frequencies for transversal and longitudinal normal modes.

Mode Number *N*	Experimental T Mode Frequency *f_T_* (Hz)	Theoretical T Mode Frequency *f_T_* (Hz)	Experimental L Mode Frequency *f_L_* (Hz)	Theoretical L Mode Frequency *f_L_* (Hz)
3	200 ± 5	208 ± 6	135 ± 5	156 ± 4
5	465 ± 5	469 ± 13	350 ± 5	374 ± 9
7	830 ± 20	783 ± 22		

## Data Availability

Data are contained within the article or Supplementary Materials.

## Supplementary Materials

The following are available online at https://www.mdpi.com/article/10.3390/mi15050634/s1, Video S1: Normal modes of vibrating drops.
